# How do United Kingdom (UK) medical schools identify and support undergraduate medical students who ‘fail’ communication assessments? A national survey

**DOI:** 10.1186/1472-6920-13-95

**Published:** 2013-07-08

**Authors:** Connie Wiskin, Eva M Doherty, Martin von Fragstein, Anita Laidlaw, Helen Salisbury

**Affiliations:** 1 Primary Care Clinical Sciences, College of Medical and Dental Sciences, University of Birmingham, Edgbaston B15 2TT, Birmingham, UK; 2 The Royal College of Surgeons in Ireland, National Surgical Training Centre, St Stephens Green, Dublin, Ireland; 3 Division of Primary Care, Community Health Sciences, University of Derby, Derby, UK; 4 Medical School, University of St Andrews, St Andrews, Fife, Scotland, UK; 5 Department of Primary Care Health Sciences, University of Oxford, Oxford, UK

**Keywords:** Clinical communication, Assessment, Survey, Support

## Abstract

**Background:**

The doctor’s ability to communicate effectively (with patients, relatives, advocates and healthcare colleagues) relates directly to health outcomes, and so is core to clinical practice. The remediation of medical students’ clinical communication ability is rarely addressed in medical education literature. There is nothing in the current literature reporting a contemporary national picture of how communication difficulties are managed, and the level of consequence (progression implications) for students of performing poorly. This survey aimed to consolidate practices for identifying and processes for managing students who ‘fail’ communication assessments across all UK medical schools.

**Methods:**

Data were collected via an email survey to all leads for clinical communication in all UK Medical Schools for the UK Council for Clinical Communication in Undergraduate Medical Education.

**Results:**

All but two participating Schools reported some means of support and/or remediation in communication. There was diversity of approach, and variance in the level of systemisation adopted. Variables such as individuality of curricula, resourcing issues, student cohort size and methodological preferences were implicated as explaining diversity. Support is relatively ad hoc, and often in the hands of a particular dedicated individual or team with an interest in communication delivery with few Schools reporting robust, centralised, school level processes.

**Conclusions:**

This survey has demonstrated that few Medical Schools have no identifiable system of managing their students’ clinical communication difficulties. However, some Schools reported ad hoc approaches and only a small number had a centralised programme. There is scope for discussion and benchmarking of best practice across all Schools with allocation of appropriate resources to support this.

## Background

The imperative to effectively teach and assess clinical communication as part of undergraduate and postgraduate medical education is recognised both at national and international levels. In the UK this has been consistently advocated by the General Medical Council [1-3] and following the Bristol enquiry it was reported that “Improving responsiveness to patients has been a goal of health policy in the United Kingdom for several decades” [4]. In 2008, and building on earlier work, The UK Council for Clinical Communication in Undergraduate Medical Education (a grouping of clinical communication leads for all UK Medical Schools, hereafter referred to as UK Council) published a national consensus statement [5] which describes a core curriculum for inclusion in medical curricula.

The literature on clinical communication is comprehensive, and has focussed on behaviours, relationships with outcomes, and methodology [6]. There is much written on assessment psychometrics [7] but the question of remediation, i.e. supporting those who do not achieve core competency, is rarely addressed in research. Reports specifically on ‘poor performance’ relating to communication are relatively ad hoc. A focus of many studies has been on variables identified as explanatory factors, for example gender [8], ethnicity [9,10], English language proficiency [11] and other wider issues of professionalism [12-14]. Communication features as a factor in studies reporting supporting students with ‘academic difficulties’ [15] and some Schools have reported specific interventions that address communication weaknesses [16]. Nothing is apparent in the current literature, however, that reports a contemporary national picture of how communication difficulties are managed, and the consequences for students (in progression terms) of performing poorly.

In the light of this, the UK Council conducted a review of current assessment and remediation of clinical communication across Medical Schools in the UK. Given the close relationship between teaching and testing, it was considered important to establish a comprehensive and current picture of assessment mechanisms across all UK Schools. It is the hope of the UK Council that this paper will share practice, and encourage consolidation of strategies for the future.

## Methods

UK Council communication leads from 33 UK Medical Schools offered to individually collate assessment data from all departments in their institutions where undergraduate communication was formally assessed. This approach reduced the risk that the survey reached just one individual or team, who may or may not be able to represent the views/initiatives of other modules on the wider degree course.

Generation of the questionnaire was a collaborative process, undertaken at a national UK Council meeting. Questions generated there were refined by a sub-group, re-circulated, and confirmed at a second meeting.

The overall survey, administered 2011, comprised two parts. The first part, A, was a quantitative review of any assessment methods employed^i^[1]. The second part, B, asked open directive questions about the nature and level of support in place for students identified through assessment as “failing”. This paper reports on the part B questions, specifically:

(1) Are there any compulsory communication assessments failure of which can prevent progression?

(2) What support is available for students failing communication, and is support systematised?

(3) What happens to students who fail compulsory communication assessments?

(4) Are poor standards of communication identified by any other processes?

Each lead received the survey by email, and streamed it to all module leads and examination teams within their organisation who summatively assessed clinical communication. Non-respondents were reminded. Data returned were merged to create a national database.

‘Yes/No’ responses were simply quantified. Where appropriate, and where participants provided more descriptive answers, comments were clustered into themes using thematic analysis [17], a methodology which allows interpretation of data while retaining a degree of theoretical freedom. “Theme’ [here] captures something important about the data in relation to the research question, and represents some level of patterned response or meaning within the data set”. Themes were developed by one of the authors [CW], and ratified by 2 members of the sub-group. Given the straightforward nature of the questions and familiar terminology in the answers there was good agreement. The number of Schools responding under each theme was subsequently quantified.

### Ethics

After consultation with the Convenor of the St Andrews Medical School Teaching and Research Ethics Committee, ethical permission was not sought for this initiative as it was deemed an internal UK Council of Clinical Communication in Undergraduate Medical Education audit of assessment practice to (a) gain a clear picture of current practice within Schools and (b) facilitate development of national standards. In the participants’ letter this was clearly stated, as was that no school would be identified, that participation was voluntary, and that any publication would be presented as a consensus of all participating Schools. All participants have reviewed the manuscript. No objections have been received.

## Results

Response rate was 88% (n = 29 of a possible 33 Schools). This represented 35 courses in total, as six additional graduate entry programmes were included.

### Are there any compulsory communication assessments failure of which can prevent progression?

It was complex to extrapolate clear answers in some cases, due to the high level of integrated skills assessed, most typically in OSCE format. “We do not have any assessments that are solely of communication since we assess content and process together in a clinical OSCE context or by other assessments based on clinical attachments” was a typical response.

In response to the initial Yes/No question, 16 Schools replied ‘Yes’ and 11 ‘No’. One school felt unable to respond, another completed the free text comment, but did not indicate a definitive answer. Clearly in some Schools failure specifically in communication did hinder course progression, and in other Schools students did progress despite failing. A number of respondents stated that assessments of communication are integrated with other clinical skills to such an extent that it is not possible to comment on the degree to which poor communication is a progression hurdle (Table 1). The awarding of ‘conditional passes’ to students passing with a communication flag, pending remediation, was noted.

**Table 1:** Communication failure as a progression hurdle

	
Clear ‘Yes’ (Failure prevents progression)	n = 6
Clear ‘No’ (Failure does not affect progression)	n = 6
Integrated assessment, but communication could impact on an overall fail	n = 9
Cannot fully say (due to level of integration in each domain)	n = 7

### What happens to students who fail compulsory communication assessments?

Six themes emerged:

1. Students ‘fail OSCEs’ rather than ‘fail communication’ which triggers a standard re-sit process. As communication is integrated into OSCEs (combined process and content scoring) communication scores are proportional. The number of overall stations needed in different examinations to gain a ‘pass’ is highly variable. Students technically can ‘pass’ the OSCE (in some cases) with very poor communication scores. (*n* = *12 Schools for this theme*).

2. Students can and do fail based on unacceptable communication scores. This can result in re-sits, and ultimately discontinuation of study. (*n* = *8 Schools for this theme*).

3. Failure at communication is not a progression hurdle, but triggers some form of intensive - usually non-compulsory - re-training. (*n* = *4 Schools for this theme*).

4. Students failing communication are referred to a committee, e.g. Academic Progress Board, who consider mitigation and can sanction termination of study. (*n* = *2 Schools for this theme*).

5. There are no compulsory communication assessments, so no consequences for failed students. (*n* = *2 Schools for this theme*).

6. Students re-sit the entire examination if the communication station is failed. (*n* = *1 school for this theme*).

### What support is available for students failing communication, and is support systematised?

58.6% of Schools operate a systematised support system (17 ‘Yes’, and 12 ‘No’). Text comments supported both responses, and were diverse. There was some overlap in the comments between the ‘yes’ and ‘no’ responders suggesting systematisation may be in the eye of the beholder: one person’s systematised individual coaching may be another person’s ad hoc response.

(A) Of those Schools who *did* identify the presence of a systematised system the following forms of this were identified:

•Individual one-on-one bespoke coaching programme, including simulation (n = 6)

•Workshop programme (n = 3)

•Mixture of individual support and remedial teaching (n = 3)

•Mixture of individual teaching and small group teaching (n = 3)

•Small group revision class (n = 1)

•One week course for OSCEs failure (not communication specific) (n = 1)

(B) Of those Schools who *did not* identify the presence of a systematised system the following forms of intervention were identified:

•Students seen individually/ad hoc (n = 4)

•Simulated patient session available (n = 3)

•“Drop in sessions”, or “on request” to record consultations watch DVDs etc. (n = 2)

•Mixed support, usually including student support services and/or welfare (n = 2)

•Ward observation (n = 1)

•Special instruction for international students (n = 1)

•Workshops in some, but not all, cases (n = 1)

One school reported that there was no known support process. Two organisations gave two responses, hence n = 14.

### Are poor standards of communication identified by any other processes?

Total numbers of responses are shown in Table 2. N > 29 as Schools indicated all relevant processes. Fitness to Practice (FTP) was the most commonly reported additional process, but with hope expressed that serious problems would be detected before this point.

**Table 2:** **Summary of non**-**assessment mechanisms for picking up communication difficulties**

**Mechanism**	**Number of schools**
FTP	14
Tutor/clinical attachments reports	12
Report cards/ flags from any staff member	6
Personal and professional development strand	3
From any part of the curriculum	3
Formative assessment	2
Simulated patient feedback	2
Critical instance reporting	1
360 degree appraisal	1
Whole year screening for communication problems (role play)	1
Not aware of any	2

## Discussion

The results overall represent the majority (29/33) of UK Medical Schools which provides opportunity to gain a national picture of communication assessment remediation for students. Limitations include the missing contribution of 4 Schools, and the difficulty in being certain that all possible stakeholders were captured within each organisation by the respondent. Respondents were responsible for remediation, and had knowledge of which module leads to contact within their Schools, but it is not impossible that other, unseen staff (e.g. on wards) might have engaged with informal and/or undisclosed ad hoc remediation, which might not have been captured by assessment teams.

Data returned relating to remediation were less than data previously collected reporting assessment method and frequency. While this might reflect lower levels of industry in providing descriptive data, the observation is consistent with weightings in published literature (where there appears more interest in pass-fail detection than in picking up students in need of remediation). This is speculatively similar for other assessed items, where more emphasis is placed on assessing than remediating, but that is not to say that there is no desire to improve.

The relationship between communication assessment and progression seems important. ‘Communication’ per se we discovered is rarely, if ever, assessed in isolation. The complex relationship between knowledge, transmission and sharing of information, professional attitudes, ethics and behaviour is acknowledged as intrinsic to holistic practice, but not fully understood or easily measured [18,19]. Communication in the current data is assessed most typically as part of an OSCE, in the context of a ‘consulting encounter’, with different weightings given to different components of the task depending on the clinical task and educational outcomes. To assess communication in isolation from the task would not be possible (communication always has some context), and selection of a non-clinical context would not help staff to appreciate the student’s ability to communicate in their professional role [20].

The validity of the holistic assessment, while appropriate vocationally and educationally, presents the conundrum of how to robustly identify and support students who ‘fail’ to communicate adequately. Respondents in this survey indicated that the overall scoring of large scale interactive exams means that it is possible for a ‘poor communicator’ to pass overall (and not receive support) if they score adequately on other components of the examination^ii^ however it is less likely that a skilled communicator could ‘pass’ overall if knowledge or safe practice competencies were not met. A cultural shift is proposed in which deficiencies in *any* aspect of professional performance are viewed equally, as already evidenced in some Schools: “Communication skills are assessed in OSCEs and mini-CX Examinations [sic] and failure at Communication Skills is viewed in the same way as failure of any other assessment”.

Although there were commonly used methods, returned data showed lack of systematic mechanisms for identifying and managing performance deficiency. Responses suggested the identification of students struggling in clinical communication was most commonly failing a summative exam such as an OSCE (which may be due to factors other than, or in addition to, communication), or was on an *ad hoc* basis by staff. However, failure to systematically identify - and therefore remediate - poor communicators may be less of a problem than first appears. Firstly, it is rare for an otherwise ‘exemplary’ student to ‘just fail communication’ – experience suggests that poor communicators struggle in a range of competencies [21]. “In practice there are very few students who fail communication in isolation”. Secondly, items on OSCE assessment schedules usually audited as ‘clinical scores’ are often fundamentally communication based, e.g. history taking, negotiating a management plan, gaining consent and explaining clinical reasoning. These highlight communication as well as clinical deficiencies, but would not show (e.g. in audit) as “communication scores”.

Some comments related to support being offered to students who “sought out help”, or were “sent to communication leads by concerned others”. While there is something to be said for initiative such a system is arguably imperfect, being dependent as it is on student insight, often lacking in poor performers. Identification of struggling students is potentially an issue for staff too. Two Schools noted the importance of staff noticing a concern (even a ‘low level’ one) having a mechanism to - eg - “flag that student to teachers who will subsequently encounter that student”. For referrals to be effective staff need awareness of (a) *how* to identify poor communication and (b) *what* to do if they do identify it. The former is a challenge. With vast numbers of clinicians encountering students on short placements not all will be skilled at - or confident in - reporting deficiency. From experience this is due to training deficit, over-focus on clinical skill, empathy with ‘nice’ students, fear of damaging progression opportunities, anxiety about ‘prejudice (e.g. international language comprehension), ignorance of referral procedures [22], or - at worst - assumption that ‘such an obvious problem is being managed elsewhere’. One respondent reported that “Staff who are less able communicators than the students cannot be relied on to mark this competency reliably”. This makes effective identification of poor performance complex, to say the least.

The dynamic of multiple staff encounters offers rich diversity of experience, and resists moderation. This must be factored into referral processes. There is no obvious answer, but that is not to say that improvement is not attainable. Twenty years ago the communication field itself was struggling for recognition as a core competency for effective medical practice [23]. That being achieved, the new gauntlet is to assess communication consistently, and manage under-achievement. *All* staff should be involved. Sometimes inappropriate behaviour manifested with, e.g., support staff is shielded from clinical seniors.

Some Schools are – encouragingly - linking communication with professionalism, which seems a positive way forward “…we have an evolving professionalism assessment (summative at the end of all clinical rotations) which has ‘relationships with patients’ and ‘communicating with colleagues’ as key domains; we also have a ‘yellow card’/cause for concern system whereby clinical teachers can - and do - alert the faculty to students who have problems…”.

There are attempts being made to support students with identified communication difficulties. Encouragingly, all but two participating Schools reported some means of support and/or remediation in the communication category. What was apparent was that while core methods repeated in the data (shared experience of e.g. role play; video) there was diversity of approach and combinations of methods, and variance in the level of systemisation adopted.

Explanations for variance are likely multi-factorial, the results of this research suggesting individuality of curricula, resourcing issues, student cohort size and methodological preferences. To a degree this mirrors the range of approaches taken to teaching and testing communication, although a combination of national institutional imperatives, increased research and interest in the speciality, the formation of a national leads group and the long-standing commitment of specialist educators has gone a long way to enhancing and standardising the student experience in these fields. In short, support for failing students needs to catch up.

The flavour of the detailed text comments is that support is relatively ad hoc, and often in the hands of a particular dedicated individual or team with an interest in communication delivery. Few Schools report robust, centralised, school level processes for managing communication referred students. Having said that, the results show clear effort from most Schools to offer some form of revision, with only one school reporting “no support”.

The type/mix of support offered does vary by School, but included coaching, one-on-one encounters, simulated patient intervention or access to resources (eg DVDs), in an attempt to help those who need it most. There was little mention in the data of pre-emptive work (possibly an artefact of the survey’s positioning), with just one comment directly related advance interventions to know remediation problems “Specific induction teaching is offered to international students who may have language/ cultural problems”.

What does not emerge is a trend for clear processes for referred students that are either widely known to students wishing to self-refer, or to clinical staff teams wanting to flag a student who appears to be in difficulty, or mandatory for all students with poor scores in this field. This may be due to a very small evidence base for the type of interventions that are effective in this situation, particularly specific to clinical communication [15].

In terms of recommendation, there were examples that might be used as models. Four Schools reported systems beyond ‘seeing the tutor’ that were linked to assessments (example 1, below) and one school reported the start of a centralised system for multi-source referrals (example 2).

Example 1 - “Students who perform badly (many fails, or lots of borderline passes) on the communication skills and history taking stations are identified by the Assessment Coordinator, and their names provided to me after an advisory interview (this interview explores reasons for poor performance e.g., not working, personal problems). These students are assessed by reviewing their performance in role play consultations, then an individualised programme of support planned, which the student agrees to (or may not agree to - but that has never happened). The support tends to include simulated consultations, lots of feedback, supervised practice on the wards (also with immediate feedback from a member of staff)”.

Example 2 - “We are working to establish a dedicated post and specialist central service to support students who are perceived as communicating poorly. Currently the system is too ad hoc to be entirely satisfactory. The [anonymised] Unit offers as much coaching and individual support as it can via a referral scheme. Referrals come via student welfare, assessment difficulty, tutor feedback and, in some cases, self-referral”.

Following up on example 2 the system is now much less ad hoc, and the team in receipt of central school finance for this purpose, including funding for clinical and non clinical staff, and simulated patients. Proactive intervention (i.e. picking up early concerns) likely makes better investment than waiting for a fail to flag a difficulty. As specified by the GMC “It is important that medical students have opportunities to seek support for any matter before it becomes a fitness to practise concern” [24]. While individual Schools doubtless have clear, individual, FTP processes, the imperative is to avoid such grave consequences for all but the most extreme cases.

Some form of ‘flagging’ system, in conjunction with centralised and well advertised resources for referral, seems a pragmatic approach to an important problem. Postgraduate referrals for poor performance in ‘non-clinical/communication’ areas encompass a complex range of attitudinal, ethical, cultural, language, team-working and management style difficulties. At this level (career progression failure) the stakes are high and the cost – emotional, personal and financial – immeasurable. UK Council leads are sharing practice to improve outcomes before the stakes are raised, but the range is apparent. Some Schools have no identifiable system, some ad hoc and multi-method approaches, and a small number a centralised programme.

## Conclusion

This survey has demonstrated that most Medical Schools have an identifiable system of managing their students’ clinical communication difficulties. However, some Schools reported ad hoc approaches and only a small number have a structured, centralised programme. The nature of integrated assessment adds challenge to measuring communication as an isolated ‘fail’ criterion, but some schools were able to report that failing to reach competence in this category could – and would – hinder progression. The majority of Schools do attach consequence to failure of mandatory communication assessment. We conclude that work on this area is being done, and that there is commonality of approach (eg methods used) emerging. It is not ideal that FTP is the most common alternative means of identifying students who struggle at communication, so given the stakes (personal and professional cost) at this stage the work reported here offers a more timely intervention.

### Recommendations

Timely intervention is possible, and requires explicit formulation. A national approach would be welcome. Practitioners who communicate poorly are a burden to their teams, at risk of complaints or progression halts, and often unhappy [25-27]. Students with consistently inadequate knowledge do not progress, so Schools might be well advised to routinely apply the same to candidates who struggle to interact. Students are likely (one might hope) to know how to access resources to ‘improve their clinical knowledge’. They arguably need guidance from their Medical Schools on areas where revision techniques may be less obvious – and this point is easy to implement now via information exchange in cases where help does exist. Centralised, resourced services that are transparent to students and staff, and not dependent the endeavour of ad hoc individuals or courses, is an aspiration. The relationship between communication and clinical outcome [28] for the patient is well known. Patient safety as well as individual and team functioning is at stake, so when considering remediation a central question is ‘can we afford not to?’.

## Endnotes

^i^ Part A responses are reported separately in the companion paper. The two reports are independent.

^ii^ N.b. This is not the case for all Schools, with a small number reporting progression hurdles based on communication (See results Table 1).

## Competing interests

The authors declare that they have no competing interests.

## Authors’ contributions

CW: Contributed to revisions of the draft survey, was involved in collecting results, qualitatively analysed results and was the main author of the complete manuscript. ED: Contributed to revisions of the draft survey, was involved in collecting results and was part of the sub group reviewing the data. Contributed to draft. MvF: Contributed to revisions of the draft survey, was involved in collecting results and was part of the sub group reviewing the data. Contributed to draft. AL: Contributed to revisions of the draft survey, was involved in collecting results (main contact). Commented on drafts of the manuscript. HS: Contributed to revisions of the draft survey, was involved in collecting results. Commented on drafts of the manuscript. All authors read and approved the final manuscript.

## Authors’ information

Connie Wiskin is a Senior Lecturer at the College of Medical and Dental Sciences, University of Birmingham. Her research specialties are interactive assessment and educational evaluation. She is Academic Lead for the Birmingham Student Selected Component of MBChB, and Co-Director of the Interactive Studies Unit.

Eva Doherty is Senior Lecturer/Clinical Psychologist at the Royal College of Surgeons in Ireland(RCSI). She is Director of the Human Factors and Patient Safety teaching and research programme at the National Surgical Training Centre in RCSI. Current research interests include personality assessment in medical education, emotional intelligence measurement and the assessment of Human Factors training programmes.

Martin von Fragstein is Associate Professor in the Faculty of Medicine & Health Sciences, University of Nottingham. He is a GP and an accomplished communication teacher. He has a specialist interest in (and consults in) substance misuse at Derby City Primary Care Trust NHS, and contributed to a national review of substance abuse teaching in undergraduate curricula.

Anita Laidlaw is a Senior Teaching Fellow and Convenor of communication skills at the Medical School, University of St Andrews, UK. Her current research interests are psychological and cognitive factors affecting communication and pedagogy.

Helen Salisbury is a GP and Honorary Senior Clinical Lecturer in the Department of Primary Care Health Sciences and Oxford University where she is medical advisor to the Health Experiences Research Group. Her current interests include the role of individual patient experience in medical education.

## Pre-publication history

The pre-publication history for this paper can be accessed here:

http://www.biomedcentral.com/1472-6920/13/95/prepub
